# Associations between exposure to perfluoroalkyl substances and body fat evaluated by DXA and MRI in 109 adolescent boys

**DOI:** 10.1186/s12940-021-00758-3

**Published:** 2021-06-28

**Authors:** Mathilde Lolk Thomsen, Louise Scheutz Henriksen, Jeanette Tinggaard, Flemming Nielsen, Tina Kold Jensen, Katharina M. Main

**Affiliations:** 1 Department of Growth and Reproduction, Copenhagen University Hospital – Rigshospitalet and Department of Clinical Medicine, University of Copenhagen, Copenhagen, Denmark; 2 International Center for Research and Research Training in Endocrine Disruption of Male Reproduction and Child Health, Rigshospitalet, University of Copenhagen, Copenhagen, Denmark; 3 Department of Environmental Medicine, Institute of Public Health, University of Southern Denmark, Odense, Denmark

**Keywords:** Perfluoroalkyl substances, Magnetic resonance imaging, Dual-energy X-ray absorptiometry, Fat percentage, Adolescence

## Abstract

**Background:**

Exposure to perfluoroalkyl substances (PFASs) has been associated with changes in body mass index and adiposity, but evidence is inconsistent as study design, population age, follow-up periods and exposure levels vary between studies. We investigated associations between PFAS exposure and body fat in a cross-sectional study of healthy boys.

**Methods:**

In 109 boys (10–14 years old), magnetic resonance imaging and dual-energy X-ray absorptiometry were performed to evaluate abdominal, visceral fat, total body, android, gynoid, android/gynoid ratio, and total fat percentage standard deviation score. Serum was analysed for perfluorooctanoic acid, perfluorooctane sulfonic acid (PFOS), perfluorohexane sulfonic acid, perfluorononanoic acid, and perfluorodecanoic acid using liquid chromatography and triple quadrupole mass spectrometry. Data were analysed by multivariate linear regression.

**Results:**

Serum concentrations of PFASs were low. Generally, no clear associations between PFAS exposure and body fat measures were found; however, PFOS was negatively associated with abdominal fat (*β* = -0.18, *P* = 0.046), android fat (*β* = -0.34, *P* = 0.022), android/gynoid ratio (*β* = -0.21, *P* = 0.004), as well as total body fat (*β* = -0.21, *P* = 0.079) when adjusting for Tanner stage.

**Conclusions:**

Overall, we found no consistent associations between PFAS exposure and body fat. This could be due to our cross-sectional study design. Furthermore, we assessed PFAS exposure in adolescence and not in utero*,* which is considered a more vulnerable time window of exposure.

**Supplementary Information:**

The online version contains supplementary material available at 10.1186/s12940-021-00758-3.

## Background

Perfluoroalkyl substances (PFASs) are both hydrophobic and lipophobic chemicals commonly used as surface treatment and lubricants [1]. Humans are mainly exposed to PFASs through ingestion of fish, water or food contaminated by grease-proof packaging, but also through consumer products such as impregnated clothes and furniture [2–4]. Dust and the indoor environment in general are also important contributors to human PFAS exposure [5]. PFASs are highly persistent and bioaccumulate [1, 5] with half-lives between 2 and 15 years [6]. PFASs are divided into different subtypes, e.g. perfluorooctanoic acid (PFOA), perfluorooctane sulfonic acid (PFOS), perfluorohexane sulfonic acid (PFHxS), perfluorononanoic acid (PFNA), and perfluorodecanoic acid (PFDA). PFASs are commonly used and detected in almost all human matrices [1, 7].

Previous studies have explored the associations between PFAS exposure and adiposity in children evaluated using anthropometric measures such as body mass index (BMI) [8–13]. BMI is a readily available measure and thus a commonly used marker of obesity but generally not considered a very sensitive measure of body fat [14], especially in children [15]. While dual-energy X-ray absorptiometry (DXA) and magnetic resonance imaging (MRI) give more accurate measurements of total and regional body fat [16–18], only four studies have used DXA [19–22] and no previous studies have used MRI.

Studies examining the impact of exposure after birth are limited, as most studies have investigated the potential effects of prenatal exposure to PFASs. Higher prenatal exposure to PFOA has been associated with increased weight at 3 months [23], higher BMI z-score at age 5 [8], higher fat mass and increased adiposity in 7- to 8-year-old girls [22, 24], higher BMI at age 12 [25] and 20 years [9]. As opposed to these findings, other studies have found that a higher prenatal PFOA exposure was associated with a lower BMI and weight in 1- and 7-year-old children [10, 26] as well as, lower BMI z-scores in children from 4 weeks to 2 years of age [27]. Another study described a lower birth weight in both boys and girls with higher PFOS exposure in utero [28]. For PFNA and PFDA, a recent Danish study found that prenatal concentrations of these chemicals were positively associated with BMI standard deviation score (SDS) in girls at 3 and 18 months [29] and a Chinese study described an association of prenatal PFNA exposure with increased body fat in boys at 5 years of age [30]. Prenatal PFOS exposure was associated with increased BMI among 5- to 9-year-old girls [11], higher BMI z-score at age 5 [8] and decreased birth weight followed by a catch-up in weight percentile at 20 months of age [31]. However, the evidence from human studies is inconsistent. Other studies did not find any associations between prenatal PFOS exposure and body fat or overweight in children at 4 years [32], 5–9 years [33], among 9-year-old girls [19] or in children age 8 for both prenatal PFOS, PFNA and PFHxS [24]. In fact, one study described that higher prenatal PFOS and PFHxS were associated with a lower BMI at age 12 [25]. An American study on prenatal PFAS exposure and adiposity at birth found that associations were both sex- and chemical-specific [34]. Overall, the effects of prenatal PFOA and PFOS on body fat and weight are ambiguous.

The existing cross-sectional studies on PFAS exposure in children generated inconclusive results [12, 13, 20, 35–43].

To our knowledge, no previous reports have investigated the association between current exposure to PFASs and body fat assessed by MRI or DXA which offer a more precise measurement of body fat than BMI. In this cross-sectional study, we studied the associations between PFAS serum concentrations and abdominal and visceral fat percentage evaluated by MRI as well as total body fat percentage, total fat percentage SDS, android versus gynoid fat and android/gynoid ratio as evaluated by DXA and MRI in 109 healthy 10- to 14-year-old boys. We hypothesised that a higher PFAS exposure was associated with a higher fat percentage.

## Methods and materials

### Study population

The Copenhagen Mother–Child Cohort is an ongoing longitudinal birth cohort. A total of 2098 mothers and their children, born between 1997 and 2001, were included in the original cohort [44]. In 2009, all children were invited to participate in a pubertal follow-up, and 1005 children (572 boys) agreed to participate (Supplementary Fig. 1).

Examinations included a clinical examination with pubertal staging and anthropometry, blood sampling and whole-body DXA. Information on socioeconomic status (SES) was obtained through questionnaires completed at the same time as the clinical examination [44]. Both parents of the child were given a score from 1 to 5 (1 = high and 5 = low) based on their educational level and current employment [44]. Children were given the score of the highest-ranking parent living with them.

In addition to the standard examination protocol, children with a minimum of five visits during childhood were invited to undergo abdominal MRI. A group of 256 children agreed to participate, and 228 children had the scan performed (119 boys, 109 girls) between January and September 2012; 31 MRI scans were later excluded (artifacts [*n* = 3], incorrect scan area [*n* = 4], different technical MRI protocols [*n* = 24, only girls]). A total of 197 children had both an MRI scan and all anthropometric measurements. Anthropometry, MRI, and DXA scans have previously been published [18]. Pubertal staging was available for 186 of the 197 participants. Both boys and girls underwent a clinical examination but due to funding restrictions PFAS concentrations were only measured in serum from boys. Therefore, all girls were excluded from this study (*n* = 114). Five boys were excluded as no serum sample was available for the analysis of PFASs, i.e. a total of 109 boys were included in the current study. To test whether the 109 boys included in our study differed from the entire cohort, we compared them to a control group of boys who underwent the same pubertal examination and DXA scan but without having an MRI during the same period (01.01.12–30.09.12). In total, 286 boys were included in this group.

### Clinical examinations

Body weight to the nearest 0.1 kg was measured using an electronic scale (SECA delta model 707, Seca, Hamburg, Germany, and Bisco Model PERS 200, Bisco, Farum, Denmark). Height was measured to the nearest mm using Harpenden Stadiometer (Holtain, Crymych, UK). All anthropometric measurements were done three times, and the mean was used. BMI was calculated as weight (kg)/height^2^ (m^2^). Pubertal development was determined using Tanner staging defined by ﻿genital stage [18]. Several investigators performed clinical examinations, including Tanner staging, and multiple workshops were held to ensure and maintain standardization [45]. A calculation of and interobserver coefficient of variation for pubertal staging was not performed.

### Magnetic resonance imaging 

This method has previously been described [18]. In brief, MRI scans were performed using a 3-Tesla MRI (Magnetom Verio; Siemens AG, Erlangen, Germany). We used a 32-channel receiver array coil. A 3D T1-weighted VIBE Dion sequence was used for fat quantification, creating images of abdominal fat and water. This was performed during a 19-s breath-hold in a transverse plane from the upper level of lumbar vertebra one to the lower level of lumbar vertebra four. Abdominal fat was assessed using automated segmentation, which was visually validated by an expert in MRI data analysis.

### Dual-energy X-ray absorptiometry 

A whole-body DXA scan was performed to assess total body fat percentage, android fat percentage, gynoid fat percentage, and the android/gynoid ratio. (Lunar Prodigy, GE Healthcare, Madison, WI, using enCORE software, version 14.10.022) The participants wore standardised light clothing, and a single trained investigator controlled all of the scans for the correct positioning [18]. Total fat percentage SDS was calculated according to national age- and sex-specific references [44, 46].

### PFAS analyses

A non-fasting blood sample was drawn from an antecubital vein between 8 am and 3 pm on the day of the visit and stored at -20 degrees Celsius. Serum concentrations of PFOA, PFOS, PFHxS, PFNA, and PFDA were quantified using online solid-phase extraction followed by liquid chromatography and triple quadruple mass spectrometry (LC–MS/MS) at the Department of Environmental Medicine, University of Southern Denmark. This method has previously been described in detail [47, 48]. The analysis includes a sample pretreatment procedure with the addition of isotope-labelled PFAS analogues (used as internal standards), protein precipitation and dilution with formic acid. The extraction was performed on a Thermo Scientific Equan MAX system, consisting of two Accela HPLC pumps (Thermo Scientific, San José, CA) and a PAL autosampler module (CTC analysis AG, Zwingen, Switzerland). The tandem mass spectrometer was a TSQ Quantum Ultra Triple Stage Quadropole with heated electrospray ionization (Thermo Scientific, San Jose, CA). A calibration curve, serum and solvent blanks and quality control samples (NIST 1958) were included in each batch of samples analysed. The accuracy of the method was controlled through regular participation in the German Quality Assessment Scheme (G-EQUAS) organized by the German Society of Occupational Medicine. Imprecision between batches was < 7%, and the limit of detection (LOD) was 0.03 ng/mL for all compounds. There were no observations below LOD for the reported PFASs.

### Statistics

Population characteristics and serum concentrations of PFASs were presented as medians as well as 25 and 75 percentiles. Correlations between different PFASs were determined using Spearman’s rank correlation coefficient. Age- and sex-specific SDS were calculated according to national references [44, 46] for height, weight, BMI and total fat percentage. Median PFAS concentrations were determined in relation to the different characteristics of the study population: Tanner stage, SES, age, BMI SDS, weight SDS, and height SDS. The Kruskal–Wallis test was used to test for differences in concentrations between these subgroups. Differences in the selected charateristics between the boys included and not included in this study were tested using the Mann–Whitney U-test for non-parametric data, the Chi-squared test for categorical variables and the students’ t-test for normally distributed variables.

PFAS serum concentrations as well as all MRI and DXA body fat measurements except for total fat percentage SDS were log transformed (base 10) to obtain a normal distribution. Linear multivariate regression analysis was performed using log transformed data to test the associations between serum PFASs and body fat percentages measured by MRI and DXA. These analyses were carried out both unadjusted and adjusted for relevant confounders. Potential confounders (Tanner stage, SES, age, birth weight SDS, and BMI SDS) were first individually tested for influencing PFAS exposure and changing the effect estimate. Thereafter, the confounders were tested in a model that included all variables except the variable that was evaluated. In the end, only the Tanner stage was included in the final model (Tanner stages 4 and 5 were combined due to low frequencies). We tested non-linear fits by adding a cubic roots PFAS in the model; this was only significant for PFHxS in 3 of the 7 fat measurements, and due to the small sample size, we did not pursue this further.

Statistical analyses were performed in SPSS, version 25.0 (IBM Corp. Released 2017. IBM SPSS Statistics for Windows, Version 25.0. Armonk, NY: IBM Corp). A statistical significance level was set at *P* < 0.05.

## Results

Population characteristics (Tanner stage, SES, age, BMI SDS, height, and weight SDS) and median PFAS concentrations are presented in Table 1. All five PFASs were detectable in all serum samples. The highest median serum concentrations were found for PFOS and PFOA (6.81 ng/mL and 2.79 ng/mL, respectively) (Table 1). Most PFASs were moderately to highly correlated (*r*: 0.50–0.84, *P* < 0.01), but weak correlations were found between PFOA and PFOS (*r* = 0.36, *P* < 0.01), PFHxS and PFOA (*r* = 0.29, *P* < 0.01), and PFDA (*r* = 0.34, *P* < 0.01) and PFNA (*r* = 0.22, *P* < 0.05) (Supplementary Table 1). Population characteristics for boys with and without a MRI scan are shown in Table 2. Only the Tanner stage was significantly different between the two groups.

**Table 1:** Characteristics of the study population and PFAS serum concentrations

	*n*	Median	25^th^-75^th^ percentiles
Age (years)	109	12.6	11.5–13.2
Tanner stage	100		
1	20		
2	33		
3	29		
4	16		
5	2		
SES	108		
1	57		
2	20		
3	36		
4	5		
5	0		
Height (cm)	109	158.3	150.9–166.7
Height SDS	109	0.11	-0.65–0.87
Weight (kg)	109	44.9	38.6–51.5
Weight SDS	109	0.01	-0.73–0.75
BMI (kg/m^2^)	109	17.7	16.5–19.8
BMI SDS	109	0.30	-0.83–0.60
Body fat assessed by MRI (%)			
Abdominal fat	109	18.9	16.5–24.8
Visceral fat	109	6.47	5.69–7.39
Body fat assessed by DXA (%)			
Total body fat	108	16.8	12.8–24.8
Total body fat SDS	108	-0.08	-0.63–0.77
Android fat	108	16.3	11.6–26.7
Gynoid fat	108	26.7	22.2–35.8
Android/gynoid ratio	108	0.6	0.51–0.78
PFAS serum concentrations (ng/mL)			
PFOA	109	2.79	2.18–3.58
PFOS	109	6.81	5.85–9.47
PFHxS	109	0.50	0.40–0.65
PFNA	109	0.92	0.73–1.17
PFDA	109	0.31	0.25–0.37

*BMI* Body mass index, *SDS* Standard deviation score, *PFAS* Perfluoroalkyl substances, *PFOA* Perfluorooctanoic acid, *PFOS* Perfluorooctane sulfonic acid, *PFHxS* Perfluorohexane sulfonic acid, *PFNA* Perfluorononanoic acid, *PFDA* Perfluorodecanoic acid, *SES* Socioeconomic status

**Table 2:** Selected characteristics of included and excluded boys

	*n*^**a**^	Included boys	*n*	Excluded boys	*P*-value^b^
		Median (25^th^-75^th^ percentiles)		Median (25^th^-75^th^ percentiles)	
Age (years)	114	12.5 (11.4–13.1)	286	12.2 (11.4–13.1)	0.372
Tanner stage	105		250		
1	23		86		
2	34		74		
3	30		52		
4	16		27		
5	2		11		**0.008**
Height (cm)	114	157.9 (150.4–166.7)	285	156.1 (148.6–163.9)	0.157
Height SDS	114	0.10 (-0.66–0.81)	285	0.01 (-0.88–0.70)	0.261
Weight (kg)	114	44.9 (38.4–50.9)	285	43.7 (37.4–50.0)	0.171
Weight SDS	114	-0.004 (-0.72–0.77)	285	-0.12 (-0.77–0.63)	0.266
BMI (kg/m^2^)	114	17.9 (16.6–19.8)	285	17.8 (16.5–19.5)	0.388
BMI SDS	114	-0.19 (-0.79–0.62)	285	-0.13 (-0.81–0.57)	0.579

Boys who participated in the pubertal follow-up were included in the present study if they also had a pubertal MRI performed, those without an MRI were excluded

^a ^Please note that five boys did not have a PFAS measurement and could thus not be included in the statistical analyses

^b ^Differences between the two groups were tested using Chi-squared test for categorical data, Mann–Whitney U-test for non-parametric data and Student’s t-test for parametric data. Significant values (*P* < 0.05) are highlighted in bold

All serum concentrations of PFASs, except for PFNA and PFDA, decreased at higher levels of Tanner stages (Table 3). PFOA and PFHxS serum concentrations significantly differed between SES group 1 and SES groups 2–4. Concentrations of all five PFASs decreased with increasing height SDS (Table 3).

**Table 3:** Serum PFAS (medians, 25^th^–75^th^ percentiles) according to Tanner stage, SES, age, and SDS for BMI, weight, and height

	*n* (%)	PFOA (ng/mL)		PFOS (ng/mL)		PFHxS (ng/mL)		PFNA (ng/mL)		PFDA (ng/mL)	
		Median	25^th^-75^th^ percentiles	Median	25^th^-75^th^ percentiles	Median	25^th^-75^th^ percentiles	Median	25^th^-75^th^ percentiles	Median	25^th^-75^th^ percentiles
Tanner stage	100										
1	20 (20)	3.05	2.58–3.58	8.57	6.70-–1.11	0.55	0.44–0.72	1.07	0.89–1.41	0.36	0.29–0.45
2	33 (33)	2.99	2.22–4.27	7.03	5.40–9.95	0.54	0.44–0.68	0.95	0.72–1.19	0.33	0.24–0.41
3	29 (29)	2.46	2.07–3.18	6.23	5.79–8.09	0.47	0.41–0.68	0.77	0.66–0.99	0.27	0.23–0.32
4 + 5	18 (18)	2.26	1.85–3.36	6.04	5.12–7.43	0.43	0.31–0.51	0.85	0.71–1.08	0.27	0.23–0.32
*P*-value*		**0.04**		**0.01**		**0.03**		**0.04**		**0.01**	
SES	108										
1	57 (52.7)	2.74	2.74–3.42	7.42	6.17–10.19	0.51	0.44–0.70	0.92	0.73–1.13	0.32	0.25–0.37
2–4	51 (47.2)	3.00	2.19–3.63	6.23	5.40–7.90	0.46	0.37–0.54	0.94	0.73–1.20	0.29	0.24–0.36
*P*-value*		0.58		**0.01**		**0.01**		0.55		0.21	
Age (years)	109										
10–11.9	36 (33.0)	3.13	2.28–4.31	6.92	6.06–8.59	0.52	0.44–0.64	0.94	0.69–1.22	0.33	0.26–0.37
12.0–12.9	41 (37.6)	2.56	2.16–3.53	7.12	5.54–10.44	0.46	0.39–0.66	0.95	0.77–1.18	0.32	0.25–0.38
13.0–15	32 (29.6)	2.68	2.00–3.30	6.63	5.85–8.71	0.48	0.41–0.66	0.82	0.71–1.04	0.28	0.25–0.36
*P*-value*		0.14		0.85		0.30		0.53		0.48	
BMI SDS	109										
≤ -1	22 (20.2)	2.77	2.17–3.13	6.90	5.97–10.53	0.47	0.43–0.56	0.96	0.68–1.20	0.27	0.25–0.40
> -1 to ≤ 0	39 (35.8)	2.79	2.09–3.31	7.83	6.03–10.0	0.51	0.41–0.70	0.95	0.74–1.14	0.34	0.27–0.37
> 0 to ≤ 1	32 (29.4)	3.08	2.27–4.99	6.56	5.87–9.29	0.52	0.42–0.67	0.91	0.73–1.42	0.31	0.23–0.40
> 1	16 (14.7)	2.60	1.89–3.62	5.86	4.97–6.73	0.41	0.32–0.60	0.78	0.73-–1.03	0.27	0.21–0.30
*P*-value*		0.39		**0.01**		0.25		0.66		**0.06**	
Weight SDS	109										
≤ -1	19 (17.4)	2.76	2.32–3.22	7.90	5.91–10.78	0.50	0.43–0.68	1.11	0.88–1.24	0.33	0.27–0.45
> -1 to ≤ 0	35 (32.1)	2.90	2.19–3.39	7.91	6.12–10.45	0.52	0.43–0.65	0.95	0.72–1.14	0.34	0.27–0.37
> 0 to ≤ 1	37 (33.9)	2.79	2.07–4.08	6.52	5.41–7.93	0.50	0.41–0.69	0.82	0.69–1.22	0.27	0.22–0.34
> 1	18 (16.5)	2.53	1.85–3.62	6.14	5.14–7.06	0.41	0.34–0.58	0.81	0.75–1.07	0.28	0.23–0.33
*P*-value*		0.79		**0.01**		0.13		0.40		**0.02**	
Height SDS	109										
≤ -1	15 (13.8)	3.17	2.69–7.14	10.11	7.12–13.03	0.68	0.54–0.85	1.11	0.91–1.27	0.36	0.31–0.52
> -1 to ≤ 0	33 (30.3)	2.84	2.24–3.44	7.91	5.99–10.12	0.51	0.43–0.65	0.95	0.69–1.21	0.34	0.26–0.40
> 0 to ≤ 1	37 (33.9)	2.92	2.19–3.62	6.23	5.57–7.48	0.47	0.40–0.65	0.90	0.73–1.07	0.29	0.25–0.34
> 1	24 (22.0)	2.37	1.89–3.06	6.32	5.31–7.13	0.43	0.38–0.54	0.79	0.63–1.09	0.26	0.21–0.33
*P*-value*		**0.06**		** < 0.01**		**0.01**		**0.09**		**0.01**	

Data are presented as medians and 25–75 percentiles. *SES* Socioeconomic status, *BMI* Body mass index, *SDS* Standard deviation score, *PFAS* Polyfluoroalkyl substances, *PFOA* Perfluorooctanoic acid, *PFOS* Perfluorooctane sulfonic acid, *PFHxS* Perfluorohexane sulfonic acid, *PFNA* Perfluorononanoic acid, *PFDA* Perfluorodecanoic acid

^*^ Differences in PFAS concentrations between subgroups were tested using Kruskal–Wallis test. *P*-values < 0.05 were considered significant and highlighted in bold

PFOS and PFDA concentrations were negatively associated with the seven body fat measurements in both the unadjusted and adjusted models, but were only significant in some cases (Table 4). In the adjusted model, PFOS and PFDA were negatively associated with both abdominal fat percentage (PFOS: *β* = -0.18, *P* < 0.05; PFDA: *β* = -0.17, *P* > 0.05), visceral fat percentage (PFOS: *β* = -0.03, *P* > 0.05; PFDA: *β* = -0.09, *P* > 0.05) evaluated by MRI, android fat percentage (PFOS: *β* = -0.34, *P* < 0.05; PFDA: *β* = -0.25, *P* > 0.05), and android/gynoid ratio (PFOS: *β* = -0.21, *P* < 0.05; PFDA: *β* = -0.21, *P* > 0.05) (Table 4). PFNA was negatively associated with both abdominal fat percentage, visceral fat percentage, android fat percentage, android/gynoid ratio and total fat percentage SDS and was positively associated with total fat and gynoid fat percentage evaluated by DXA; however, none of the associations were significant in either the unadjusted or adjusted models. There were positive but non-significant associations between PFOA and PFHxS and all fat measurements except for the android/gynoid ratio in both the unadjusted and adjusted models. However, when adjusting for Tanner stage, the association between PFHxS and total fat percentage SDS turned negative (Table 4).

**Table 4:** Associations between serum PFAS concentrations and six different MRI and DXA body fat measures

	**MRI** *β* (95% CI)		**DXA** *β* (95% CI)				
	**Log abdominal fat %**	**Log visceral fat %**	**Log total fat %**	**Log android fat %**	**Log gynoid fat %**	**Log android/gynoid ratio**	**Total fat % SDS**
**Log PFOA**							
Unadjusted	0.04 (-0.13, 0.20)	0.04 (-0.08–0.15)	0.09 (-0.12, 0.31)	0.08 (-0.19, 0.35)	0.12 (-0.05, 0.28)	-0.37 (-0. 18, 0.10)	0.47 (-0.60, 1.53)
Adjusted	0.07 (-0.12, 0.25)	0.002 (-0.13–0.13)	0.09 (-0.15, 0.33)	0.08 (-0.22, 0.39)	0.11 (-0.08, 0.30)	-0.02 (-0.18, 0.13)	0.49 (-0.71, 1.69)
**Log PFOS**							
Unadjusted	-0.20 (-0.37, -0.04)*	-0.03 (-0.14–0.09)	-0.20 (-0.42, 0.02)	-0.34 (-0.61, -0.07)*	-0.09 (-0.27, 0.08)	-0.24 (-0.38, -0.11)*	-1.10 (-2.18, -0.02)*
Adjusted	-0.18 (-0.37, -0.003)*	-0.03 (-0.16–0.09)	-0.21 (-0.44, 0.02)	-0.34 (-0.64, -0.05)*	-0.13 (-0.31, 0.05)	-0.21 (-0.36, -0.07)*	-1.12 (-2.28, 0.04)
**Log PFHxS**							
Unadjusted	0.001 (-0.16, 0.16)	0.02 (-0.10–0.13)	0.05 (-0.15, 0.26)	0.03 (-0.24, 0.29)	0.08 (-0.08, 0.24)	-0.06 (-0.20, 0.08)	0.18 (-0.86, 1.21)
Adjusted	0.03 (-0.15, 0.20)	0.02 (-0.11–0.14)	0.01 (-0.22, 0.23)	-0.01 (-0.30, 0.28)	0.01 (-0.16, 0.19)	-0.02 (-0.17, 0.13)	-0.04 (-1.17, 1.10)
**Log PFNA**							
Unadjusted	-0.06 (-0.22, 0.01)	-0.01 (-0.13–0.10)	0.01 (-0.21, 0.22)	-0.07 (-0.34, 0.20)	0.06 (-0.11, 0.22)	-0.12 (-0.26, 0.02)	-0.01 (-1.08, 1.06)
Adjusted	-0.04 (-0.22, 0.14)	-0.05 (-0.17–0.08)	0.03 (-0.21, 0.26)	-0.05 (-0.35, 0.25)	0.07 (-0.11, 0.25)	-0.12 (-0.27, 0.03)	0.12 (-1.05, 1.29)
**Log PFDA**							
Unadjusted	-0.19 (-0.37, -0.01)*	-0.05 (-0.18–0.07)	-0.11 (-0.35, 0.12)	-0.26 (-0.55, 0.04)	-0.01 (-0.20, 0.17)	-0.25 (-0.4, -0.09)*	-0.72 (-1.90, 0.47)
Adjusted	-0.17 (-0.37, 0.04)	-0.09 (-0.23–0.05)	-0.12 (-0.38, 0.15)	-0.25 (-0.58, 0.08)	-0.04 (-0.24, 0.16)	-0.21 (-0.37, -0.05)*	-0.70 (-2.00, 0.60)

Associations were tested in multiple linear regression models, and the table shows the resulting effect estimates (*β*). For fat percentages and android/gynoid ratio, these express the change in the log value of these five outcomes per log unit increase in serum PFAS concentration. Tanner stage was included as a covariate in the adjusted models

*PFAS* Perfluoroalkyl substances, *MRI* Magnetic Resonance Imaging, *DXA* Dual X-ray absorptiometry, *CI* Confidence interval, *PFOA* Perfluorooctanoic acid, *PFOS* Perfluorooctane sulfonic acid, *PFHxS* Perfluorohexane sulfonic acid, *PFNA* Perfluorononanoic acid, *PFDA* Perfluorodecanoic acid, *SDS* standard deviation score

*P*-values < 0.05 were considered significant and highlighted with an asterisk (*)

Explanation on how to interpret effect estimates using an example PFOA vs. total fat percentage by DXA: When PFOA serum concentration increases by a factor 10, the median value of total fat percentage increases by 23% (10^0.09^ = 1.23)

## Discussion

To our knowledge, this is the first cross-sectional study exploring the associations between serum concentrations of PFASs and abdominal as well visceral fat measured by MRI and total body fat, android as well as gynoid fat, the android/gynoid ratio and total fat percentage SDS measured by DXA in 10- to 14-year-old boys. Generally, we found no clear and consistant associations between serum concentrations of PFASs and fat measurements evaluated by DXA or MRI. However, higher serum concentrations of PFOS were associated with lower abdominal fat percentage, android fat percentage and android/gynoid ratio. This is congruent with the findings of an American study in teenage boys (12 to 19 years old) in which boys with the highest serum PFOS concentration had a significantly lower BMI compared to the lowest exposed group [39]. Furthermore, a British cross-sectional study found that higher exposure to PFOS, FPOA, PFHxS, and PFNA was associated with a lower BMI z-score in 5-year-old children [49]. As we cannot offer any biological explanation for why PFOS would reduce body fat, unknown confounders can influence the results. Since PFASs have long half-lives we assume that the current exposure is congruent with the previous exposure [6], but as our study is cross-sectional, we cannot assess causality.

Although non-significant, we found that an increase in PFOA concentration was associated with an increase in all body fat measurements except the android/gynoid ratio which decreased. In accordance with this, an American study found a dose–response association between PFOA and the odds of being overweight or obese in children aged 12 to 18 years [13]. Similarly, a Danish study found significant associations between increased PFOA and PFOS exposure and a higher insulin and triglyceride concentration among overweight children between 8 and 10 years of age [36]. However, when assessing normal-weight children, ﻿no significant associations were found for BMI, insulin or triglyceride concentration [36]. Our population only included a few obese participants (defined by BMI SDS > 2, *n* = 2), so we are not able to sufficiently assess the effects in obese children.

A previous American study described that higher PFOA exposure was significantly associated with decreased fat percentage in 6- to 9-year-old girls [50], but the association changed direction when examining the girls again when they were 12–13 years old [41]. Another study investigating PFASs and obesity in children at age 12 while also stratifying for sex, found a consistent association between PFAS levels and obesity in girls, but not in boys [21]. Girls may be more sensitive to PFASs, and associations may differ between sexes, by chronological age or by puberty stage.

PFOS concentrations measured in our study were somewhat lower than what has been reported by other studies on children and teenagers with blood sampling between 2003 and 2011 [37, 39, 51, 52]. This may have contributed to the fact that we overall did not find consistent associations between exspoures and measures of body fat. As serum samples in our study are from 2012, the lower exposure levels may be explained by the legislative changes on the use of PFASs enforced in 2008 [53]. However, our serum concentrations of PFOA and PFNA were similar to levels found in some other studies on children [37, 51, 52].

In 1997, Timmermann et al. performed blood sampling with median concentrations of PFOS evaluated to 41.5 ng/mL, six times higher than in our study, and PFOA levels of 9.3 ng/mL, three times higher than in our study [36]. Twelve years later, the children were re-examined. At this point, serum PFOS concentrations at the age of 8–10 years were positively associated with BMI at around 15 years of age. This association was not found for PFOA [37]. ﻿The discrepancy between our findings and Domazet et al. may thus be explained by their considerably higher median PFOA and PFOS concentrations and the longitudinal follow-up. Within the same population, Domazet et al. also investigated PFOS serum concentrations at age 15 but found no changes in BMI from age 15 to 21 years [37]. In a study where PFASs was measured both prenatally and at age 12, only prenatal PFOA and PFHxS levels were associated with a greater risk of obesity at age 12 [21]. This is in accordance with another study where PFOS were measured prenatally and at age 5, where only prenatal PFOS exposure was associated with an increase in BMI z-score [49]. The available evidence suggests that the human fetus may be more vulnerable to PFAS exposure in utero than children or adolescents are to postnatal exposure.

For PFOS, PFDA and PFNA we found that an increased serum concentration was associated with a decrease in the investigated body fat measurements which is in accordance with the findings from a recent longitudinal study on 316 2-year-old infants [35]. Moreover, the study by Lee et al. showed that higher serum concentrations of all five PFASs were associated with decreased weight gain from birth to 2 years of age, but this was only significant for PFNA [35]. The same tendency was found in a Danish study, in which PFNA, PFDA, and PFHxS were insignificantly though consistently associated with a lower fat mass in 9-year-old children [40]. A study from Norway showed that PFHxS exposure was associated with obesity age 15–19 [12]. In contrast, an American cross-sectional study from 2020 found that the current PFHxS serum concentration was insignificantly associated with a decrease in BMI z-score in 3- to 11-year-old boys [42]. However, PFHxS concentrations were twice as high as in our study. Overall, we found that effects on body composition differed between PFASs subtypes. This observation is supported by a recent American study [20] which investigated associations between plasma PFAS levels and body composition in adolescents aged 11–16 years using DXA. They found that while PFOS and PFHxS were associated with less truncal fat, less total fat mass and decreased BMI, PFDA and PFNA were associated with increased visceral fat mass [20]. In our study, we found similar results although we saw a modest decrease in visceral fat percentage with higher PFDA and PFNA serum concentrations. In general, consistency between studies is limited. This may be explained in part by variation in exposure levels, study designs and populations, different and sometimes inaccurate measurements of body fat. However, this is also suggests that other confounding factors such as lifestyle, genetic susceptibility and exposure to other environmental checmials may modify effects of PFASs on body composition.

To our knowledge, no previous studies have assessed body fat by both MRI and DXA for studies related to PFAS exposure. Because it is accessible and affordable, BMI seems to be the most commonly used measure. However, it may not be a suitable proxy for body fat, especially in children [15]. With MRI and DXA, it is possible to achieve highly accurate measurements of fat percentages and to determine fat distribution through the assessment of regional fat, e.g. abdominal (or android) fat. Higher amounts of abdominal fat are associated with an increased risk of cardiovascular disease [54]. Previous studies investigating fat distribution in children and adolescents have shown that android fat is significantly and independently associated with less favorable plasma lipids, blood pressure, and ventricular mass. Therefore, fat distribution is a more important risk factor for cardiovascular disease than overall adiposity [55]. This is in accordance with a longitudinal study showing that android and gynoid fat have a stronger association with cardiovascular disease risk factors compared to body fat mass in both obese and normal weight boys during puberty [56]. Another study found the android/gynoid ratio to be associated with increased insulin resistance and dyslipidimea, and they saw a strong relationship between android/gynoid ratio and cardiovascular risk in boys aged 7–13 [57]. In our study we assessed associations between PFAS concentrations and specific fat tissue measures. We could not support our hypothesis that increased PFAS concentrations were associated with an increased amount of fat tissue in general or in a change of the android/gynoid ratio. In the study by Li et al., prenatal PFOA and PFHxS were associated with an increased and PFOS and PFNA levels were associated with a decreased cardiometabolic risk at age 3 [58]. However, in a Spanish study prenatal PFAS exposure was not associated with cardiometabolic risk in children at 4 and 7 years of age [59]. Overall, we found no clear associations between android/gynoid fat ratio and PFASs. This may be due to the fact that our study population was small and predominantly representing normal-weight children. As we did not assess specific cardiovascular risk factors, we cannot comnpare our data with these previous results.

As opposed to many other persistent chemicals, PFASs are not lipophilic. Therefore, PFASs are bound to proteins [1] and found in many different tissues, e.g. serum, kidney, and liver [7, 60]. Very little is known about the biotransformation, metabolism, and biodegradation of PFASs [61], but they contain strong carbon-phosphate bonds that exclude them from normal degradation pathways [1, 62].

A major strength of this study is highly specific and accurate assessment of both total and regional fat done by MRI and DXA. Additionally, PFAS levels were measured by LC–MS/MS, which is considered a state-of-the-art method [48]. However, our study also has some limitations. Firstly, as the study population was small, homogenous, and not representative of the general population, there is a risk of selection bias and low statistical power. SES was generally high, and very few participants were obese. Boys with more extreme BMIs might have declined participation in this longitudinal follow-up. Mainly resourceful families with high SES could have accepted invitation, which influences lifestyle factors such as diet and exercise, which were not assessed in our study. Secondly, we also did not assess simultaneous exposure to other environmental chemicals with potential effects on body compositon. PFAS serum levels were unknown to the participants (and the examiners) and therefore were unlikely to have affected their participation or examination results, thereby leading to a potential non-differential misclassification or underestimating our findings. Thirdly, since our hypothesis was tested on many different fat measurements, chance findings due to multiple comparisons should be considered. Finally, there were several examinators performing the pubertal examinations, which may have lead to a degree of misclassification. However, in general, the Tanner stage of puberty had very little impact on the direction of size of estimates. The cross-sectional nature of the study makes reverse causation a possible explanation of our findings.

## Conclusions

In this cross-sectional study of healthy normal-weight teenage boys exposed to low levels of PFASs, we found no consistent associations between PFAS exposure and any measures of body fat composition despite highly sensitive assessment of body composition by DXA and abdominal MRI. This suggests that postnatal PFAS exposure at low levels may have less adverse effects with respect to body fat accumulation than in utero exposure.

## Supplementary Information

**Additional file 1: Supplementary Figure 1**. Flowchart for the present study. **Supplementary Table 1**. Heat map showing Spearman correlation coefficients between the five PFASs.

## Data Availability

The datasets generated and/or analysed during the current study are not publicly available due to GDPR regulations but are available from the corresponding author on reasonable request and with permission from the data protection agency.

## Abbreviations

BMI: Body mass index
DXA: Dual-energy X-ray absorptiometry
HPLC: High-performance liquid chromatography
LC–MS/MS: Liquid chromatography and triple quadrupole mass spectrometry
LOD: Limit of detection
MRI: Magnetic resonance imaging
PFASs: Perfluoroalkyl substances
PFOA: Perfluorooctanoic acid
PFOS: Perfluorooctane sulfonic acid
PFHxS: Perfluorohexane sulfonic acid
PFNA: Perfluorononanoic acid
PFDA: Perfluorodecanoic acid
SDS: Standard deviation score
SES: Socioeconomic status
