# Supplementary material for: Associations between exposure to perfluoroalkyl substances and body fat evaluated by DXA and MRI in 109 adolescent boys
Source: Environ Health. 2021 Jun 28;20:73. doi: 10.1186/s12940-021-00758-3 (PMC8244201; doi:10.1186/s12940-021-00758-3)
Supplement: Supplementary file 1 — Additional file 1: Supplementary Figure 1. Flowchart for the present study. Supplementary Table 1. Heat map showing Spearman correlation coefficients between the five PFASs. [file 12940_2021_758_MOESM1_ESM.docx]

**Supplementary Figure 1:** Flowchart for the present study

Excluded

2098 children in the Copenhagen Mother-Child cohort

1005 children (46.9%) agreed to participate in pubertal follow-up

(boys, n = 572; girls, n = 433)

≤ 5 previous visits

256 children agreed to participate in MRI (boys, n = 131; girls, n = 125)

14 non-compliant (claustrophobia)

14 cancellations

3 major artefacts

4 inaccurate sequences

24 different MRI scanning protocol

197 children with MRI

(boys, n = 114; girls, n = 83)

Missing

Excluded

No PFAS measurement, all girls (n = 83)

No blood sample available for PFAS analysis (n = 5)

Excluded

109 boys

Missing

**Supplementary Table 1:** Heat map showing Spearman correlation coefficients between the five PFAS

|  | PFOA | PFOS | PFHxS | PFNA | PFDA |
| --- | --- | --- | --- | --- | --- |
| PFOA | 1 |  |  |  |  |
| PFOS | 0.36** | 1 |  |  |  |
| PFHxS | 0.29** | 0.61** | 1 |  |  |
| PFNA | 0.71** | 0.57** | 0.22* | 1 |  |
| PFDA | 0.53** | 0.69** | 0.34** | 0.84** | 1 |

Correlations were were tested by Spearman rank correlation test.

*P*-values < 0.05 were considered significant, and P-value < 0.001 were considered highly significant, both were highlighted with an asterisk (**P* < 0.05, ***P* < 0.001)
